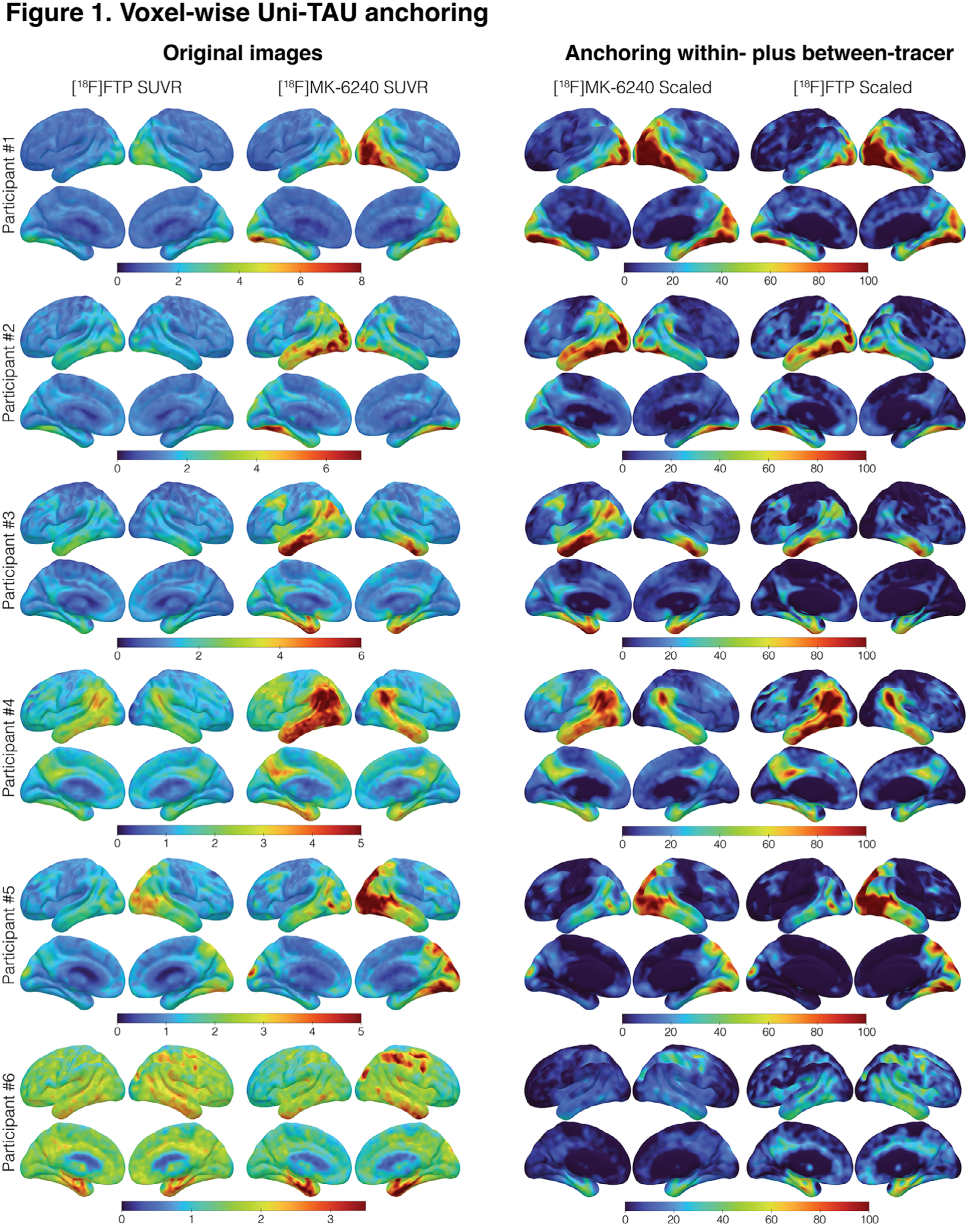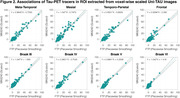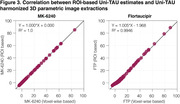# Creating Universal Tau PET Scale (Uniτ) Parametric Images – The HEAD Study

**DOI:** 10.1002/alz.093141

**Published:** 2025-01-09

**Authors:** Guilherme Povala, Guilherme Bauer‐Negrini, Bruna Bellaver, Firoza Z Lussier, Livia Amaral, Pamela C.L. Ferreira, Quentin Finn, Bruno Zatt, Dana Tudorascu, William J. Jagust, William E Klunk, Val J. Lowe, David N. soleimani‐meigooni, Hwamee Oh, Belen Pascual, Brian A. Gordon, Pedro Rosa‐Neto, Suzanne L. Baker, Tharick Ali Pascoal

**Affiliations:** ^1^ University of Pittsburgh, Pittsburgh, PA USA; ^2^ Houston Methodist Research Institute, Houston, TX USA; ^3^ Universidade Federal de Pelotas, Pelotas Brazil; ^4^ Lawrence Berkeley National Laboratory, Berkeley, CA USA; ^5^ Department of Radiology, Mayo Clinic, Rochester, MN USA; ^6^ Memory and Aging Center, Weill Institute for Neurosciences, University of California, San Francisco, San Francisco, CA USA; ^7^ Brown University, Providence, RI USA; ^8^ Washington University in St. Louis School of Medicine, St. Louis, MO USA; ^9^ Translational Neuroimaging Laboratory, The McGill University Research Centre for Studies in Aging, Montréal, QC Canada

## Abstract

**Background:**

The HEAD study focuses on collecting an extensive dataset from various tau‐PET tracers, aiming to establish robust anchor values, which are essential for harmonizing tau‐PET measurements. Here, we aim to showcase the capability of converting 3D tau‐PET images into a common scale using the Universal Tau‐PET Scale, Uniτ (tau), and to use these 3D images to subsequently obtain ROIs as needed.

**Methods:**

We assessed 185 individuals across the aging and AD spectrum from the HEAD study, with [^18^F]Flortaucipir and [^18^F]MK‐6240 tau‐PET tracers. Tau‐PET SUVR images were standardized to a common 8mm FWHM, using the inferior cerebellar gray matter as the reference region. We generated Uniτ Tau‐PET 3D images using a single formula based on Meta‐temporal parameters in two steps: first, within‐tracer anchoring based on young (<25 years) and cognitively impaired individuals; second, between‐tracer anchoring using a piecewise transformation from [^18^F]Flortaucipir to [^18^F]MK‐6240, with smoothing at the piecewise inflection point. Subsequently, we extracted mean Uniτ values from these 3D images for key ROIs, including meta‐temporal, mesial, temporo‐parietal, frontal, and Braak stages III‐VI. Finally, we correlated Uniτ values across ROIs between the two tracers to evaluate the accuracy of estimates from the voxel‐wise transformation.

**Results:**

The original SUVR images present large differences between the two tau‐PET tracers (Figure 1). However, upon applying the Uniτ piecewise transformation with smoothing to all brain voxels, we were able to reasonably harmonize these images to the Uniτ scale, substantially reducing visual variability. Notably, the mean Uniτ values for the ROIs extracted from these harmonized 3D tau‐PET brain images demonstrated a high level of association between the two tracers (Figure 2). Furthermore, estimates generated from ROIs or extracted from our 3D parametric Uniτ model yielded identical estimates (Figure 3).

**Conclusion:**

The strong associations between tracers after directly harmonizing 3D images to Uniτ scale using the piecewise transformation with smoothing, underscore the effectiveness of the proposed method. This approach provides a reliable and standardized way to compare tau‐PET data across different tracers. Our results indicate the feasibility of harmonizing 3D tau‐PET images without relying on pre‐established ROIs, overcoming the limitation of restricting the analysis to only few brain regions.